# Muscle acetylcholine receptor high resolution structures: insights into development and autoimmune disease

**DOI:** 10.1063/4.0000884

**Published:** 2025-10-27

**Authors:** Huanhuan Li, Minh Pham, Jinfeng Teng, Kevin O’Connor, Colleen Noviello, Ryan Hibbs

**Affiliations:** 1 University of California San Diego, La Jolla, CA, USA; 2 Yale University, New Haven, CT, USA

## Abstract

All voluntary muscle contraction is triggered by acetylcholine (ACh) binding to its ionotropic receptors (AChRs) at neuromuscular junctions. However, the structure of human muscle AChR, the receptor switch during muscle development, and receptor-based mechanisms underlying human muscle weakness remained unknown, mostly due to the lack of an effective method for the mammalian/human muscle receptor preparation. Here, we first developed a method to purify micrograms of muscle receptor protein from kilograms of bovine muscle (beef) to yield high-resolution structures of both fetal and adult acetylcholine receptors, then used electrophysiology to understand development of the neuromuscular junction. To study the autoimmune disease myasthenia gravis (MG), we next developed a method to express and purify the full- length functional human adult muscle AChR from a stable cell line and determined high-resolution cryo-EM structures of the human AChR in complex with a panel of six MG patient-derived autoantibodies. These antibodies were previously characterized for the classical mechanisms of autoantibody-mediated pathology: receptor blockade, internalization, and complement activation. Our findings provide an MG autoantibody epitope map for the human muscle AChR, revealing unexpected diversity in antibody binding sites. Complementary electrophysiological and binding assays further elucidate the distinct binding mechanisms underlying pathogenic receptor inhibition. I will talk about methodology of native and recombinant receptor preparations in the oral section and present the mechanistic stories regarding to receptor switch during muscle development and myasthenia gravis in poster section.

